# Transperineal Ultrasound-Guided Endoscopic Sinusotomy for Recurrent Perianal Sinus in Crohn Disease

**DOI:** 10.14309/crj.0000000000001769

**Published:** 2025-07-21

**Authors:** Partha Pal, Mohammad Abdul Mateen, Rajesh Gupta, Manu Tandan, D. Nageshwar Reddy

**Affiliations:** 1 Medical Gastroenterology, Asian Institute of Gastroenterology, Hyderabad, India

## CASE REPORT

A 30-year-old woman with Crohn disease (Montreal classification A2L3B2p) who had prior history of sigmoid colectomy, ileocecal resection, and 2 surgical fistulectomies for perianal fistula currently presented with recurrent discharge for last 2 months at a prior fistulectomy site. She had been in stable remission on adalimumab 40 mg every other week and azathioprine (2 mg/kg) for the last 2 years.

**Figure 1.**
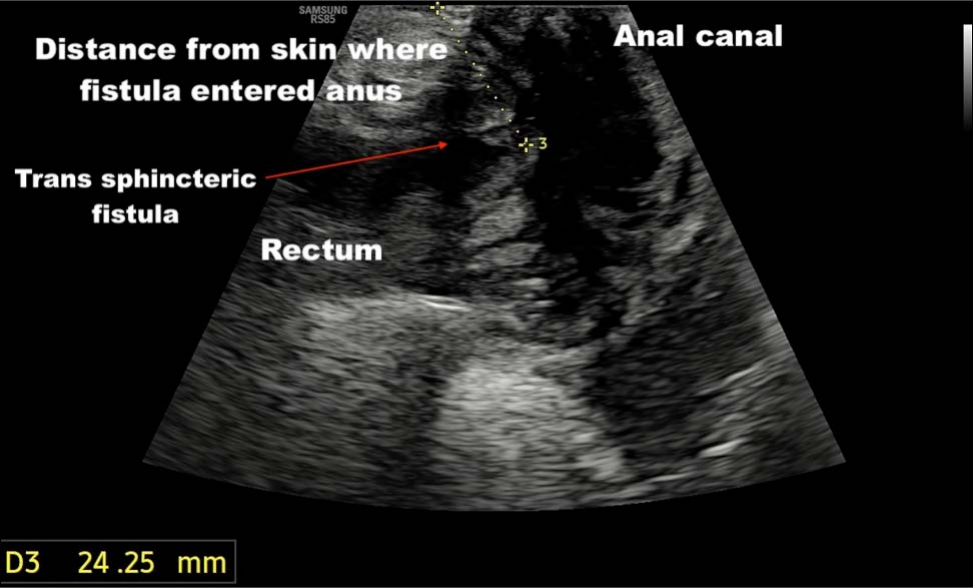
Transperineal ultrasound showing trans-sphincteric fistula tract originating from the 6 o'clock position of the anal canal.

**Figure 2.**
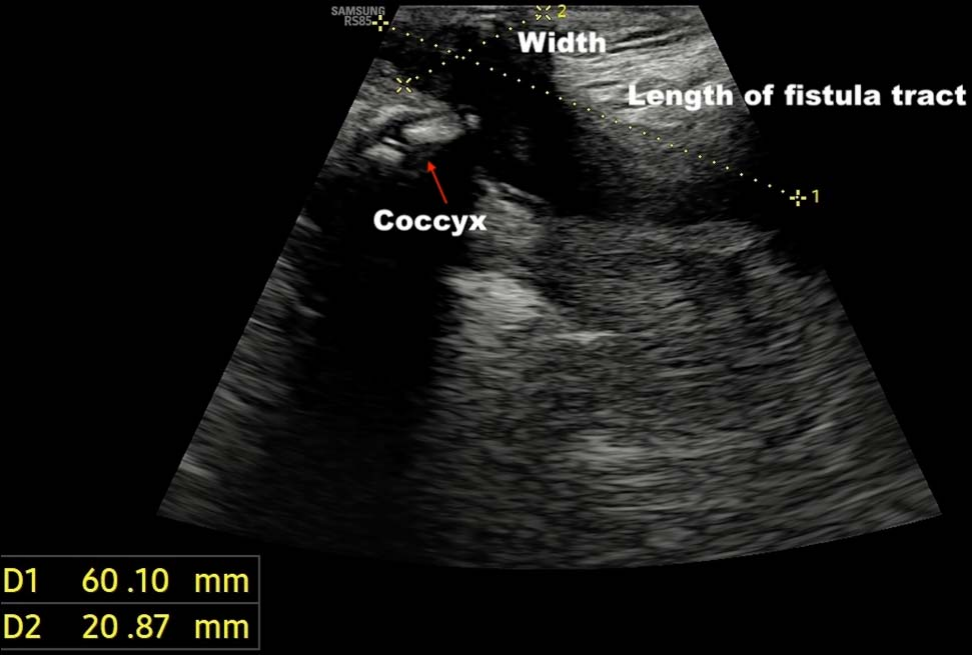
Transperineal ultrasound image revealing posterior extension of the fistulous tract reaching up to the level of the coccyx.

**Figure 3.**
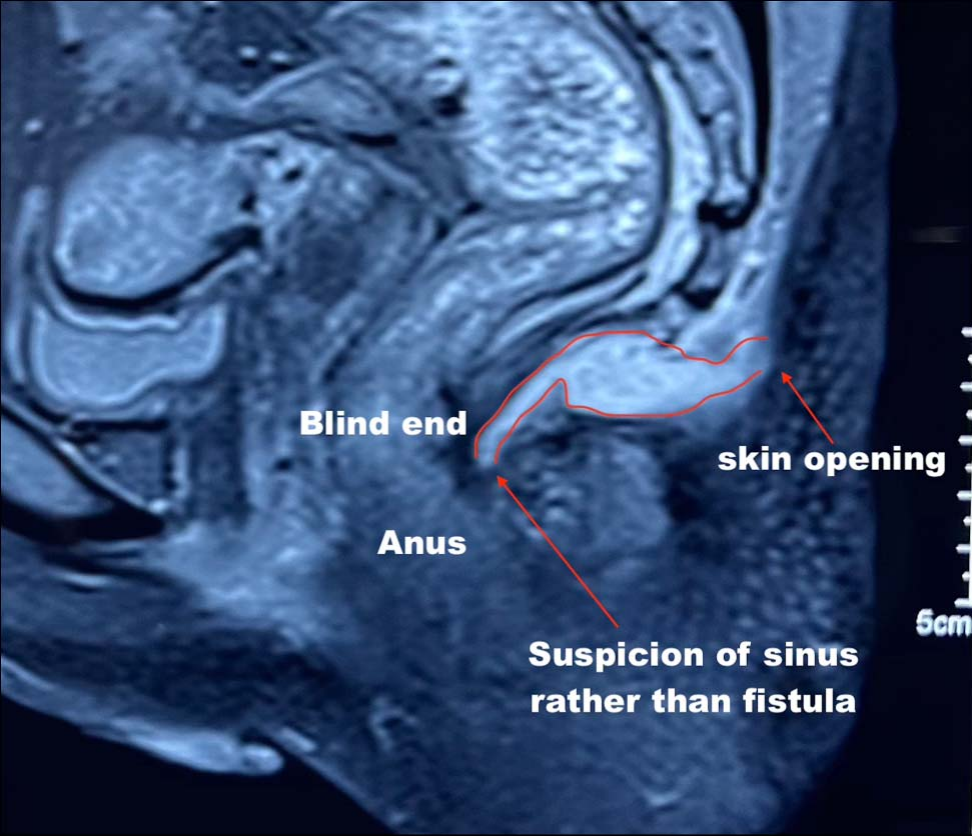
Magnetic resonance imaging pelvis demonstrating sinus tract without clear visualization of an internal opening.

**Figure 4.**
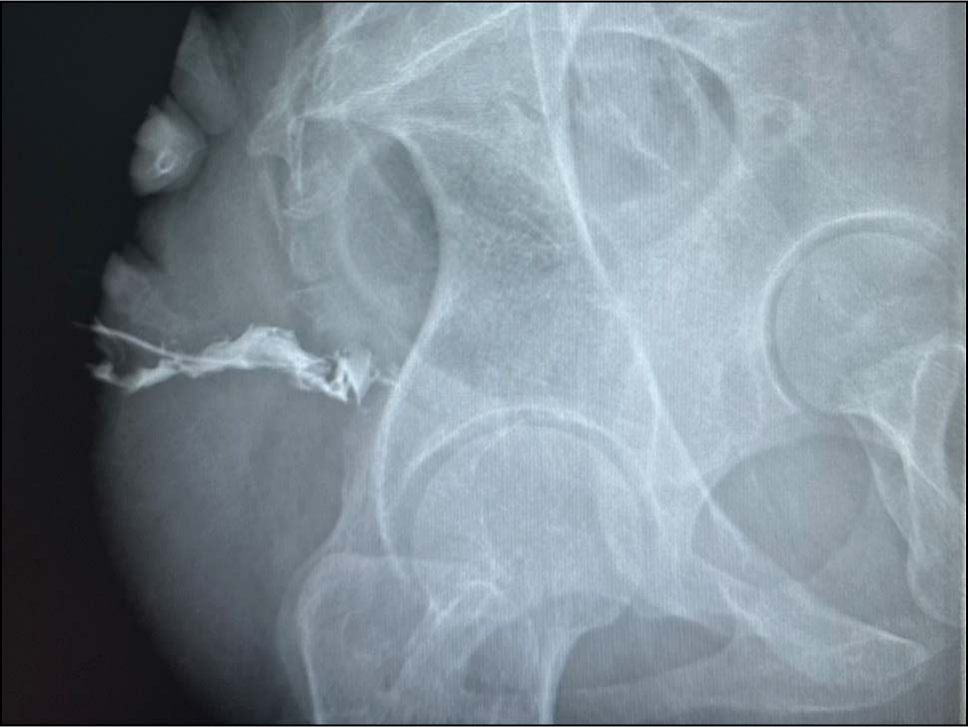
Fistulogram using iodinated contrast confirming sinus tract without communication to rectum.

**Figure 5.**
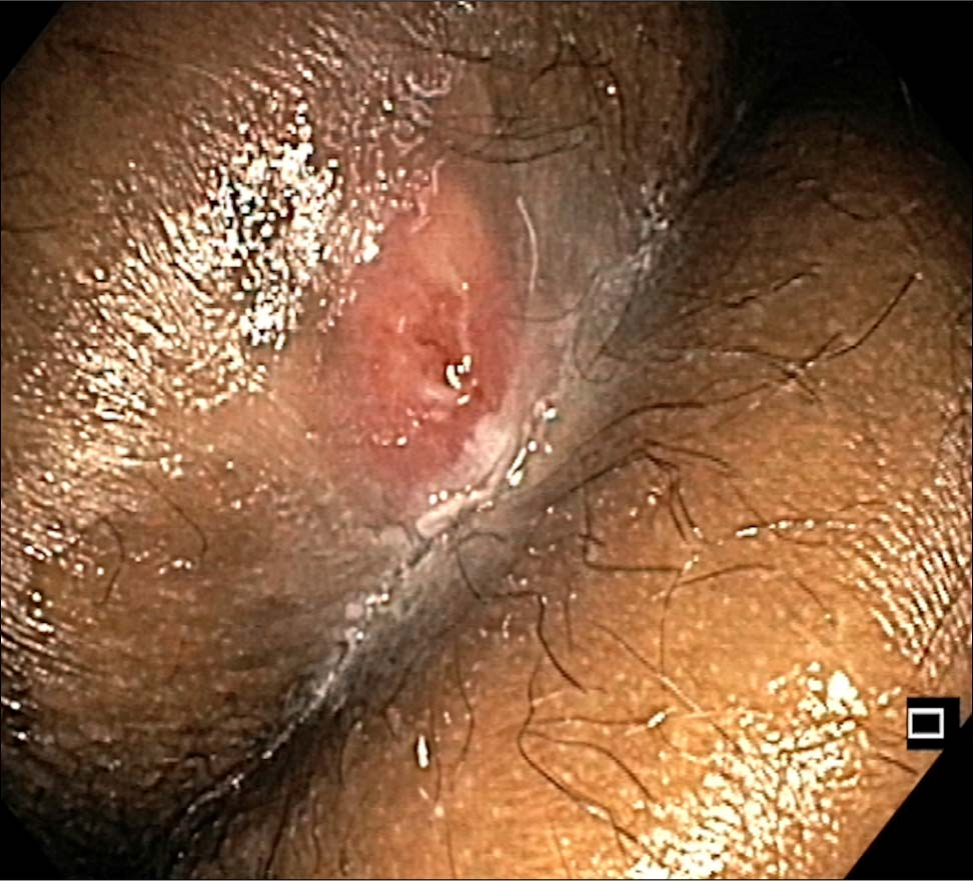
Endoscopic view during sinusotomy using needle knife.

**Figure 6.**
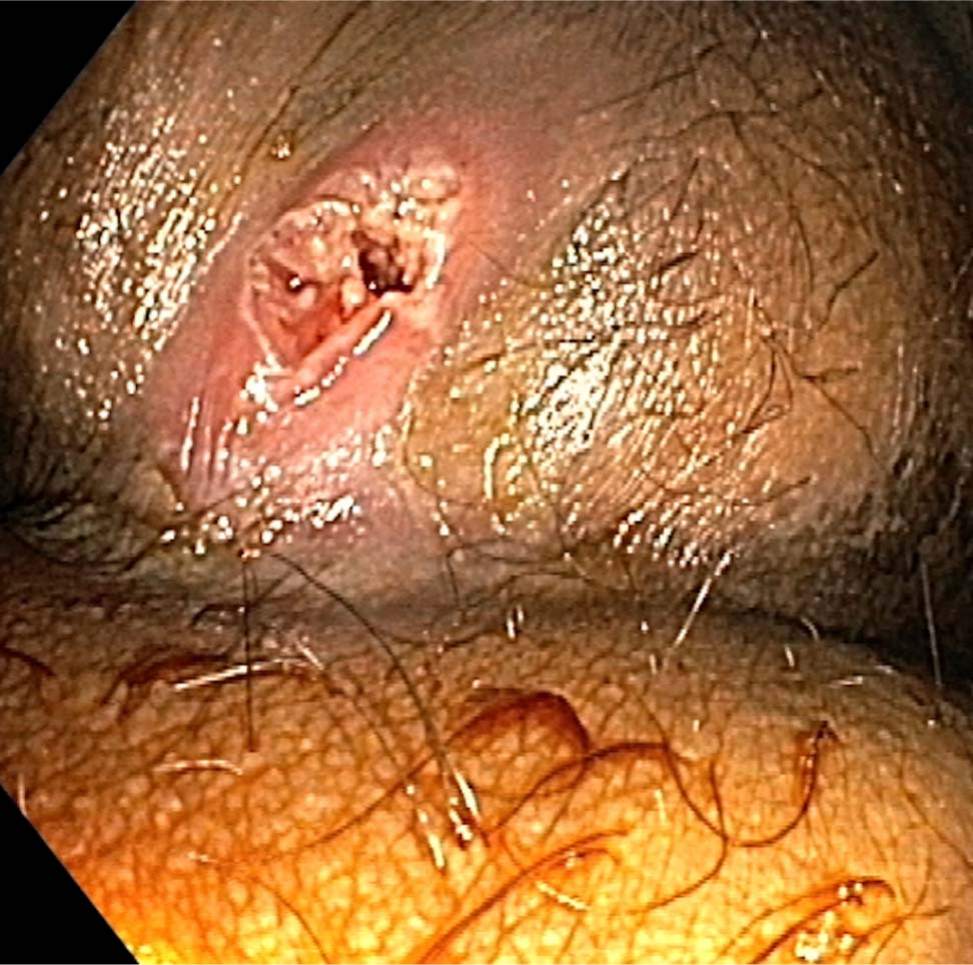
Post-procedure image showing decompressed sinus tract after completion of endoscopic fistulotomy.

Point of care transperineal ultrasound showed trans-sphincteric fistula extending from 6 o'clock of anal canal extending posteriorly up to the level of coccyx (Figures 1–2). While escalation of therapy was planned, an endoscopic seton was attempted to establish effective drainage using a stiff guidewire with hydrophilic tip (Jag wire, Boston Scientific, Marlborough, MA). Colonoscopy showed normal sigmoid anastomosis and few superficial ulcers at ileal inlet, however, failed to identify any internal opening of fistula. The guidewire could not be passed across external opening of fistula with suspicion of angulated tract or sinus. Hence, a magnetic resonance imaging (MRI) pelvis was done, which showed the possibility of a sinus tract as internal fistula opening could not be seen properly (Figure 3). Fistulography injecting iodinated contrast Omnipaque 350 (Iohexol, 350 mg I/mL, GE Healthcare, Shanghai, China) confirmed the diagnosis of sinus as no contrast was draining into rectum (Figure 4). After multidisciplinary meeting, a minimally invasive endoscopic sinusotomy was done as a day-care procedure under conscious sedation. Endoscopic sinusotomy was done using a needle knife (Olympus Medical Systems Corporation, Tokyo, Japan) making radial incisions using free hand technique with electrocautery settings of Endocut I (effect 3, cut duration 1, cut interval 3) (ERBE Elektromedizin GmbH, Tübingen, Germany), to lay open the tract and facilitate drainage (Figures 5 and 6). The procedure was completed in 20 minutes without complications, and the patient was discharged the same day. Adalimumab therapy was escalated to a weekly regimen along with azathioprine (1 mg/kg: dose reduced due to leucopenia), and ciprofloxacin was continued for 8 weeks. At the 3-month follow-up, the patient remained symptom-free, without any fistula discharge.

This case highlights the role of transperineal ultrasound in precise mapping of perianal fistulas, facilitating targeted, sphincter-preserving, minimally invasive endoscopic fistulotomy.^1^ The combination of real-time ultrasound guidance with adjunctive magnetic resonance imaging and fistulography enhanced diagnostic accuracy and ensured optimal treatment planning. Endoscopic fistulotomy is a safe, effective, and well-tolerated procedure, providing a day-care alternative to conventional surgery with faster recovery and favorable long-term outcomes in Crohn's-related perianal fistula and sinus.^2,3^

## DISCLOSURES

Author contributions: P. Pal conceptualized the case, performed the procedure, and drafted the initial manuscript. MA Mateen conducted the transperineal ultrasound and contributed to manuscript revision. R. Gupta, M. Tandan, and DN Reddy critically reviewed the manuscript and provided expert input. All authors approved the final version of the manuscript. P. Pal is the article guarantor.

Financial disclosure: None to report.

Informed consent was obtained for this case report.

## ABBREVIATIONS:

ERBE: Erbe Elektromedizin GmbH
GE Healthcare: General Electric Healthcare
MA: Massachusetts
MRI: Magnetic Resonance Imaging
TPUS: Transperineal Ultrasound
